# Drug Off-Target Effects Predicted Using Structural Analysis in the Context of a Metabolic Network Model

**DOI:** 10.1371/journal.pcbi.1000938

**Published:** 2010-09-23

**Authors:** Roger L. Chang, Li Xie, Lei Xie, Philip E. Bourne, Bernhard Ø. Palsson

**Affiliations:** 1 Department of Bioengineering, University of California San Diego, La Jolla, California, United States of America; 2 Skaggs School of Pharmacy and Pharmaceutical Sciences, University of California San Diego, La Jolla, California, United States of America; 3 San Diego Supercomputer Center, University of California San Diego, La Jolla, California, United States of America; Fox Chase Cancer Center, United States of America

## Abstract

Recent advances in structural bioinformatics have enabled the prediction of protein-drug off-targets based on their ligand binding sites. Concurrent developments in systems biology allow for prediction of the functional effects of system perturbations using large-scale network models. Integration of these two capabilities provides a framework for evaluating metabolic drug response phenotypes *in silico*. This combined approach was applied to investigate the hypertensive side effect of the cholesteryl ester transfer protein inhibitor torcetrapib in the context of human renal function. A metabolic kidney model was generated in which to simulate drug treatment. Causal drug off-targets were predicted that have previously been observed to impact renal function in gene-deficient patients and may play a role in the adverse side effects observed in clinical trials. Genetic risk factors for drug treatment were also predicted that correspond to both characterized and unknown renal metabolic disorders as well as cryptic genetic deficiencies that are not expected to exhibit a renal disorder phenotype except under drug treatment. This study represents a novel integration of structural and systems biology and a first step towards computational systems medicine. The methodology introduced herein has important implications for drug development and personalized medicine.

## Author Summary

Pharmaceutical science is only beginning to scratch the surface on the exact mechanisms of drug action that lead to a drug's breadth of patient responses, both intended and side effects. Decades of clinical trials, molecular studies, and more recent computational analysis have sought to characterize the interactions between a drug and the cell's molecular machinery. We have devised an integrated computational approach to assess how a drug may affect a particular system, in our study the metabolism of the human kidney, and its capacity for filtration of the contents of the blood. We applied this approach to retrospectively investigate potential causal drug targets leading to increased blood pressure in participants of clinical trials for the drug torcetrapib in an effort to display how our approach could be directly useful in the drug development process. Our results suggest specific metabolic enzymes that may be directly responsible for the side effect. The drug screening framework we have developed could be used to link adverse side effects to particular drug targets, discover new uses for old drugs, identify biomarkers for metabolic disease and drug response, and suggest genetic or dietary risk factors to help guide personalized patient care.

## Introduction

Despite the advantages gained from drug therapy in medicine, drug development has historically presented an expensive and frequently perplexing challenge for researchers. Identifying useful drug targets for treating disease and matching them to chemical compounds that can elicit the desired effect through drug-target interaction has been the paradigm for the drug development process in the era of molecular medicine. However, this approach has yielded many failed drug treatments and an incomplete understanding of the consequences of treatments for human health, even with drugs that have made it to market and been prescribed for decades. Two major contributing factors that confound individual molecular target-based drug discovery are drug off-target binding and the lack of systems-level understanding of drug response [1]. Adopting a new, systems-based approach to drug development is therefore a desirable goal in the era of systems medicine.

The growing wealth of omics data offers a valuable opportunity for novel approaches in systems medicine but also presents significant challenges for data integration [2]. Increasingly sophisticated computational approaches are being developed to analyze and manipulate omics data in order to gain a greater understanding of complex biological systems. An algorithm for identifying and comparing ligand binding sites on protein structures [3] was recently employed to predict drug off-target binding sites across the proteome [4]. Such a tool offers unique capabilities for drug development by providing a comprehensive survey of uncharacterized drug targets that may participate directly in drug response, which is likely to be important as polypharmacology interactions suggest that drug promiscuity is a predominant property of existing drugs [5].

Biological systems exhibit redundant pathways and synergistic effects conferring a robustness of phenotype when confronted with external stimuli. As a result, multi-target drugs are generally more clinically efficacious than single-target drugs. These facts highlight the critical importance of studying polypharmacology in a systems level context [6]. The increasing use of genome-scale metabolic network models for a variety of applications [7], [8] has established this research platform as a promising means for studying the emergent properties of complex systems. The published applications of metabolic models for drug development have thus far focused on identifying drug targets for antibacterial treatment in such pathogens as *M. tuberculosis* [9], [10], *S. aureus* [11], [10], *H. pylori*, and *E. coli* [10]. However, the human metabolic network reconstruction (Recon 1) [12] and developed context-specific metabolic modeling algorithms [13], [14] permit human-centered *in silico* drug studies. Integrating these structural bioinformatics and human system modeling techniques for application in drug development represents a first computational step into the era of systems medicine. As an example of this integrative approach, the results of protein off-target prediction for the drug torcetrapib [4], a cholesteryl ester transfer protein (CETP) inhibitor, were evaluated in the context of a model of renal metabolism.

CETP inhibitors are intended to treat patients at risk for atherosclerosis and other cardiovascular diseases by raising high-density lipoprotein cholesterol (HDL-C) and lowering low-density lipoprotein cholesterol (LDL-C) [15]. Torcetrapib was withdrawn from phase III clinical trials after a substantial investment of labor and capital due to its observed side effect of fatal hypertension in some patients [16]. It has since been of great interest to elucidate the cause of this side effect in order to avert such failures in the future and to better define the potential of CETP inhibitors for treatment [17]. Subsequent studies have provided evidence in favor of the hypothesis that the cause of this side effect was not due directly to the mechanism of HDL-C and LDL-C regulation via CETP inhibition [18]. Instead, it has been suggested that the hypertensive side effect may result from uncharacterized drug off-target effects [17]. Two other CETP inhibitors are now under clinical trial, anacetrapib [18] and JTT-705 [19]. Thus far, studies have not indicated the same risk of hypertension associated with the latter two drugs; however, these studies have been limited to relatively small patient groups lacking in diversity and over relatively short-term treatment. Even if these alternative CETP inhibitors do not carry the same adverse side effects, it is still of value to future drug development to determine the exact mechanism of torcetrapib's adverse action. It has been suggested that off-target effects of torcetrapib lead to increased activity of the renin-angiotensin-aldosterone-system (RAAS) and thereby hypertension [4], [20], but a recent review of the published CETP inhibitor clinical studies [21] concludes that the effect on blood pressure is most likely independent of the increase in aldosterone. Currently the exact cause of the hypertensive side effect of torcetrapib remains to be unambiguously identified.

The predicted torcetrapib off-targets include many metabolic enzymes and metabolite transport proteins. Although there are several mechanisms involved in regulating blood pressure that may be responsible for the hypertensive side effect, one possible mode is the renal regulation of blood pressure via metabolite reabsorption and secretion. The kidneys are the primary organs that filter the blood and therefore are strong contributors to maintaining a normotensive state even independent of RAAS function. Thus a model of renal metabolism was developed as the system context in which to analyze torcetrapib off-targets and predict drug response phenotypes. The two best-supported causal off-targets predicted in this study are prostaglandin I2 synthase (PTGIS), due to decreased capacity for renal prostaglandin I2 (PGI2) secretion, and acyl-CoA oxidase 1 (ACOX1), due to decreased capacity for renal citrate and amino acid reabsorption. Four other predicted off-targets are also predicted to impact amino acid, glucose, citrate, or bicarbonate reabsorption. As well, the model predicts no effect on renal reabsorption or secretion for a number of other predicted off-target metabolic proteins.

The goal of this study is not only to provide new insight into the torcetrapib problem but also to reveal the theoretical implications that this computational systems medicine platform has for drug development and personalized medicine. Characterizing the influence that genetic variation has in determining drug response phenotypes has been recognized as a crucial goal for the future of drug development [22]. To this end, the renal model was also used to analyze metabolic disorders resulting from genetic deficiencies and to identify those deficiencies that may pose additional risks for drug treatment in select individuals.

Although many of the predictions generated by this approach are supported by clinical and other experimental evidence that describe the impact of loss of function for predicted causal off-targets and genetic deficiencies, the full set of exact metabolic mechanisms of drug action predicted by our model remain to be completely validated. While this is seen as a limitation of this study, it also offers a number of opportunities to experimentally evaluate promising hypotheses that, if validated, will lead to significant advancements in developing CETP inhibitors for treatment and novel insight into certain renal disorders.

## Results

### Renal Metabolic Model

**Figure 1:**
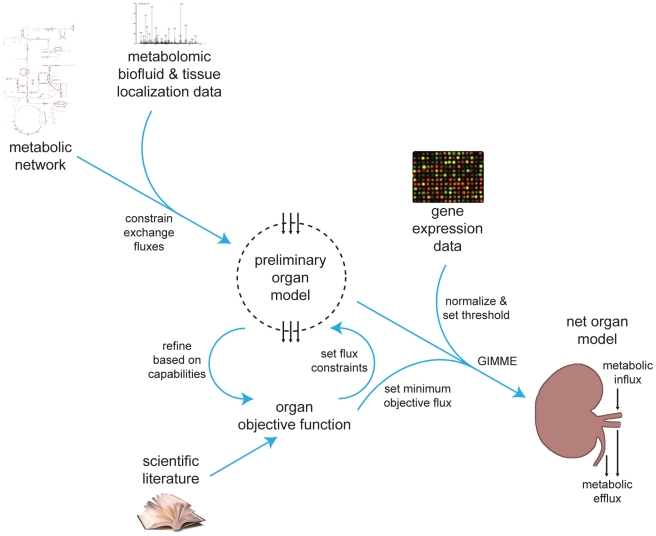
Context-specific organ metabolic modeling. Preliminary constraints were imposed upon metabolite exchange fluxes of the full metabolic network based on coordinated experimental detection of transportable metabolites both in the organ tissue and the biofluids processed by the organ. Metabolites detected in both biofluid and organ were assumed freely exchangeable in the model, and the remainder of the metabolite exchanges were tentatively constrained to zero. Organ physiology literature was reviewed to compile an objective function consisting of the metabolic functions of the organ. Each function was tested for compatibility with the preliminary model. Metabolite exchange, transport, and demand reactions required to achieve some functions were added to the network, and exchange fluxes for objective metabolites were directionally constrained in accordance with the literature. Functions not compatible with the model were removed from the overall objective function. The objective function was then integrated with gene expression data obtained from an organ tissue sample to derive a net, context-specific metabolic organ model representing the metabolic exchange between the organ and the rest of the body and the metabolic reactions that take place within the organ to achieve this exchange.

The approach for context-specific organ modeling proposed in this study (see Materials and Methods and Figure 1) yielded a renal metabolic model capturing functions of the kidney for reabsorption and secretion (Table 1). Many components of the renal objective function are factors known to be relevant determinants of blood pressure. However, there is currently incomplete knowledge about the exact role that some of these components play in blood pressure regulation. Calcium reabsorption, for example, leads to vasoconstriction in kidney glomeruli through the action of L-type and N-type calcium ion channels [23] suggesting a resulting increase in blood pressure if this mechanism applies across all vascular tissues. Calcium reabsorption also leads to an inhibition of renal sodium reabsorption in the proximal tubule [24] suggesting a blood pressure lowering effect consistent with the observation that increased dietary calcium also lowers blood pressure [25]. This highlights the complexity of the effect certain renal reasborptions have on blood pressure. Nevertheless, the many components accounted for in the renal objective function enabled explicit predictions about how system perturbations such as drug treatment and genetic deficiencies affect the kidney's ability to regulate the small molecule content of the blood.

**Table 1:** Renal objective function.

Exchange	Class	Metabolite	Abbreviation	Relation to Blood Pressure	Reference
secretion	hormones	Prostaglandin I2	PGI2	vasodilation	[60], [61]
		Prostaglandin D2	PGD2	vasodilation	[61]
		Calcitriol	-	lowers blood pressure, Ca2+ reabsorption	[62]–[65]
	urea	Urea	-	water/ion counter current system regulating osmolality	[66]
	cyclic amp	Cyclic AMP	cAMP	important for vaso-dilation/constriction	[67]
	urate	Urate	-	uknown, but secreted	[68]
	tryptamine	Tryptamine	-	uknown, but secreted	[67]
absorption	water	Water	H2O	determinant of blood pressure, ion absorption	[66], [69], [70]
	ions/electrolytes	Phosphate	-	determinant of blood pressure	[71], [72]
		Sodium	Na+	determinant of blood pressure	[70]
		Calcium	Ca2+	determinant of blood pressure	[23]–[25], [63], [73], [74]
		Chloride	Cl-	determinant of blood pressure	[75]–[77]
		Protium	H+	determinant of blood pressure	[78], [79]
		Potassium	K+	determinant of blood pressure	[25], [75]
		Bicarbonate	HCO3-	determinant of blood pressure	[78]
	carboxylates	Acetate	-	unknown, but reabsorbed	[80]
		Citrate	-	effects sodium reabsorption	[81]
		Oxalate	-	effects sodium reabsorption	[82]
	glucose	D-Glucose	-	effects sodium reabsorption	[80], [83]–[85]
	amino acids	L-Alanine	Ala	associated reduction of hypertension/vasodilation	[80], [85], [86]
		L-Arginine	Arg	associated reduction of hypertension/vasodilation	[80], [85], [86]
		L-Asparagine	Asn	associated reduction of hypertension/vasodilation	[80], [85], [86]
		L-Aspartate	Asp	associated reduction of hypertension/vasodilation	[80], [85], [86]
		L-Cysteine	Cys	associated reduction of hypertension/vasodilation	[80], [85], [86]
		L-Glutamine	Gln	associated reduction of hypertension/vasodilation	[80], [85], [86]
		L-Glutamate	Glu	associated reduction of hypertension/vasodilation	[80], [85], [86]
		Glycine	Gly	associated reduction of hypertension/vasodilation	[80], [85], [86]
		L-Histidine	His	associated reduction of hypertension/vasodilation	[80], [85], [86]
		L-Isoleucine	Ile	associated reduction of hypertension/vasodilation	[80], [85], [86]
		L-Leucine	Leu	associated reduction of hypertension/vasodilation	[80], [85], [86]
		L-Lysine	Lys	associated reduction of hypertension/vasodilation	[80], [85], [86]
		L-Methionine	Met	associated reduction of hypertension/vasodilation	[80], [85], [86]
		L-Phenylalanine	Phe	associated reduction of hypertension/vasodilation	[80], [85], [86]
		L-Proline	Pro	associated reduction of hypertension/vasodilation	[80], [85], [86]
		L-Serine	Ser	associated reduction of hypertension/vasodilation	[80], [85], [86]
		L-Threonine	Thr	associated reduction of hypertension/vasodilation	[80], [85], [86]
		L-Tryptophan	Trp	associated reduction of hypertension/vasodilation	[80], [85], [86]
		L-Tyrosine	Tyr	associated reduction of hypertension/vasodilation	[80], [85], [86]
		L-Valine	Val	associated reduction of hypertension/vasodilation	[80], [85], [86]
	oligopeptides	L-Carnosine	-	unknown, but reabsorbed	[66], [87], [88]
		Glutathione	GSH	unknown, but reabsorbed	[54]

**Figure 2:**
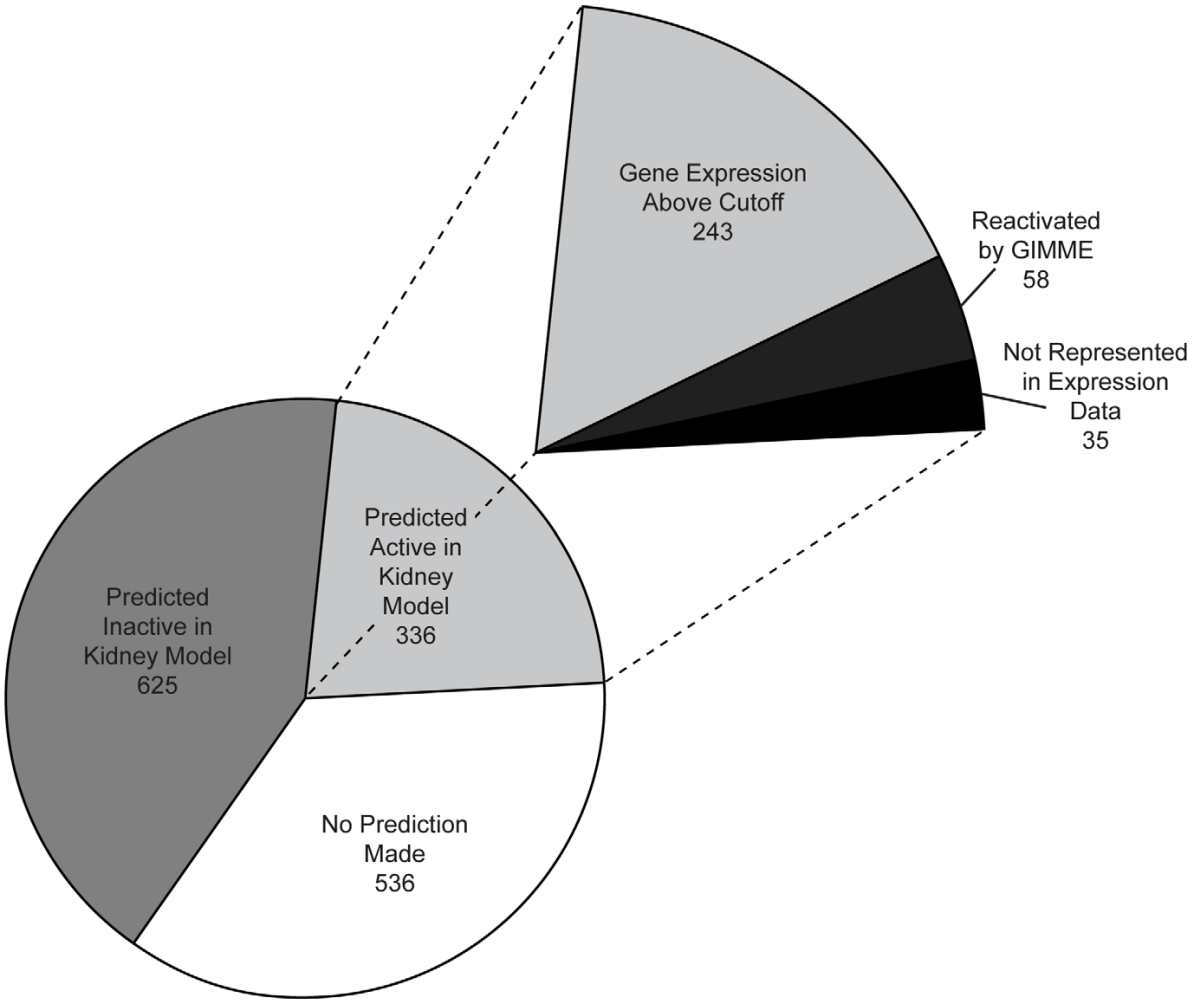
Summary of gene activity predictions in the full kidney model. The pie chart at bottom represents the Recon1 gene activity predictions resulting from deriving the kidney model. Genes predicted inactive are those genes with no associated active reaction fluxes in the kidney model. Genes for which no activity prediction was made are those associated with active reaction fluxes in the kidney model but either are not represented in the gene expression data or were not determined as the gene whose expression level is most limiting for any associated reaction through evaluation of GPR Boolean rules with respect to gene expression data. The slice at top represents genes predicted active in the kidney model.

The kidney model included 336 explicitly predicted active metabolic genes (Table S1) that met criteria for activity as summarized in Figure 2. The majority, 243 genes, satisfied the gene expression significance threshold (see Materials and Methods), although the activity of 58 genes was predicted despite expression values below the threshold. These genes were activated by the GIMME algorithm [13] to optimally achieve the renal objectives while remaining minimally inconsistent with gene expression data and may represent post-transcriptionally upregulated genes. The other 35 genes were predicted to be active without penalty since no corresponding probesets existed on the microarray upon which the transcriptomic data was obtained. Since many of these genes participated in optimal pathways for achieving renal objectives, it is projected that experimental measurement would confirm their expression if performed.

**Figure 3:**
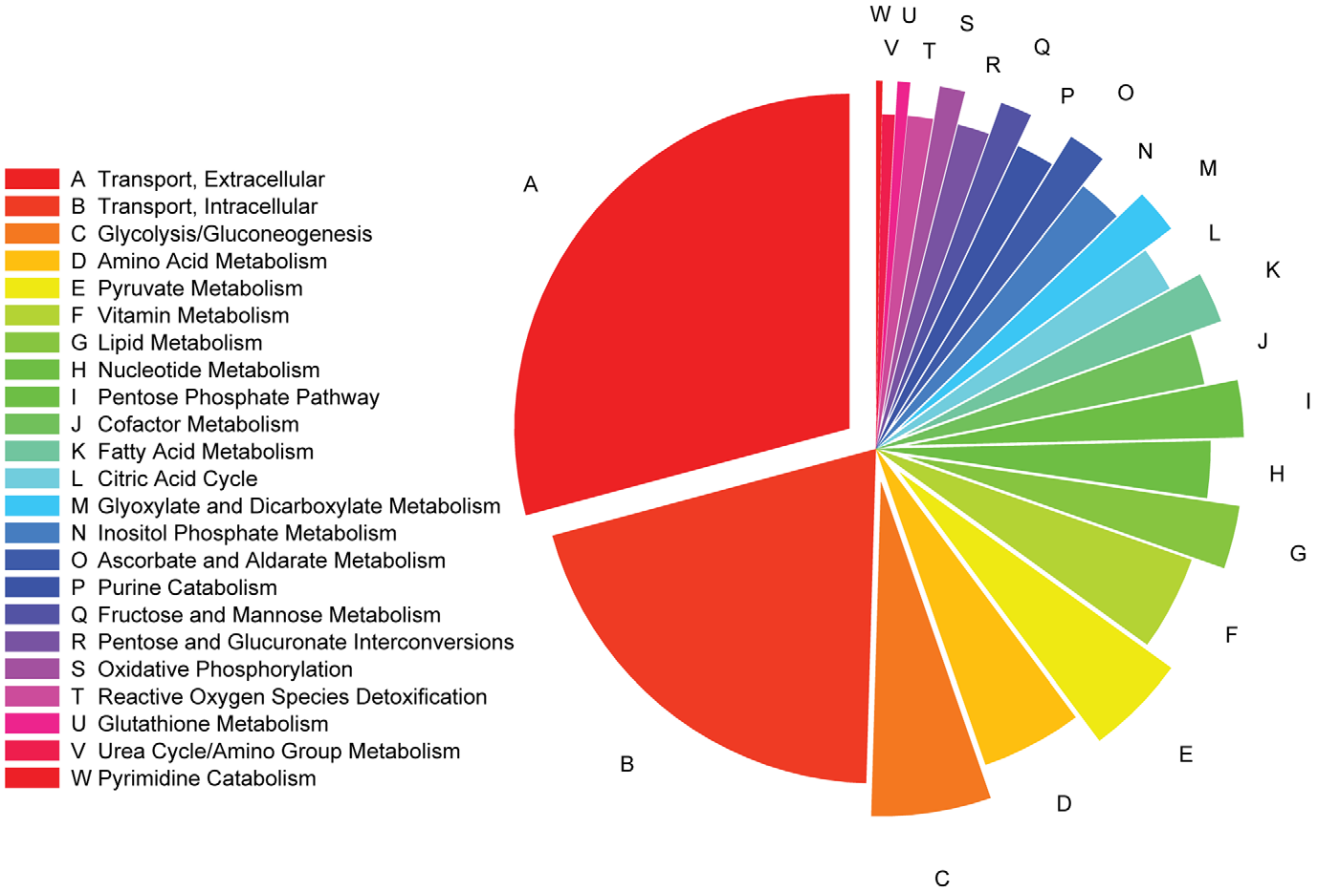
Reduced kidney model subsystem distribution. The distribution of metabolic reactions predicted to be active in the reduced kidney model with respect to broad metabolic subsystem categories is shown. The distribution excludes objective function, exchange, and demand reactions used to perform simulations in the model.

The active reactions in the model reflect both the possible pathways by which the kidney can achieve the specified renal objectives as well as other functions supported by the gene expression data. The model included 1587 active reactions (Table S2), excluding model-based reactions such as objective functions, exchanges, and demands. Of these active reactions, 333 comprised a single connected sub-model accounting for all pathways which could possibly support the specified renal objectives. We refer to this sub-model as the reduced kidney model (see Table S1 and Table S2 for the contents of the reduced model and Dataset S1 for the actual model in SBML format). It should be noted that because the reduced model included all reactions that can carry flux in support of the renal objectives, it had the exact same effective flux state solution space as the full renal model. The reduced kidney model reactions spanned a broad range of metabolic subsystems (Figure 3). The largest subsystem consisted of plasma membrane-spanning transport reactions, which is expected given that this model captured renal filtration and secretion functions. The second largest subsystem represented intracellular transport, signifying the importance of interaction among sub-cellular compartments in renal function including the cytosol, endoplasimic reticulum, Golgi apparatus, and mitochondria. A significant proportion of the other active subsystems in the reduced kidney model were involved directly in the metabolism of components of the renal objective function including carbohydrate, amino acid, vitamin, lipid, carboxylate, and glutathione metabolism as well as the urea cycle. These permitted the indirect reabsorption of metabolites as well as the required synthetic pathways for renal secretions.

### Causal Drug Off-Targets

**Figure 4:**
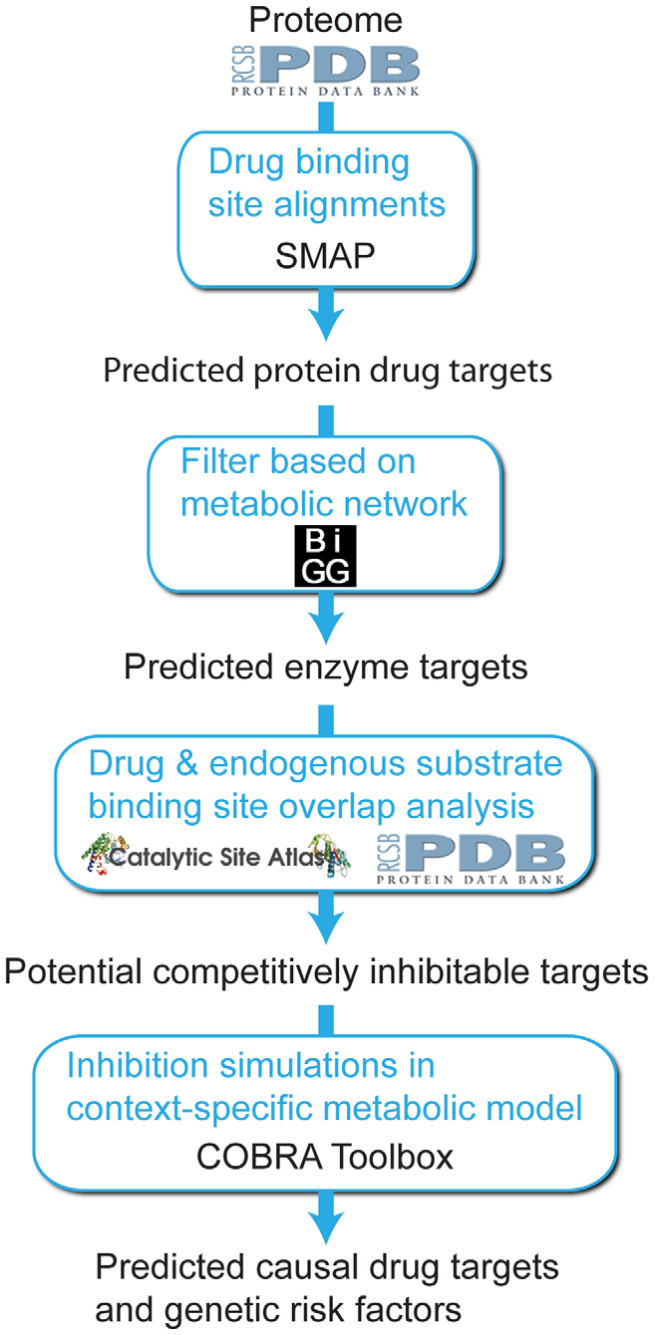
Identifying causal genes for drug response phenotypes and metabolic disorders. First, the human proteome was screened to identify off-target drug-binding sites. The resulting list of putative off-targets was filtered to focus on just metabolic proteins. Then, for each predicted metabolic off-target, the endogenous functional sites were compared to the predicted drug-binding site to identify overlap. Off-target proteins for which overlapping binding sites were identified were considered to be competitively inhibitable by the drug at the overlapping endogenous functional sites. The functional consequences of such inhibitions were then tested in an appropriate context-specific metabolic model. All possible individual gene knockouts were also simulated to predict genetic disorders that lead to functional deficiencies either alone or in combination with drug treatment. Those off-targets whose inhibition impacted model function represent causal off-targets predicted to be associated with the drug response phenotype, and the gene knockouts that impacted model function represent genetic risk factors for metabolic disorders, which may lead to amplification of the drug response phenotype.

**Figure 5:**
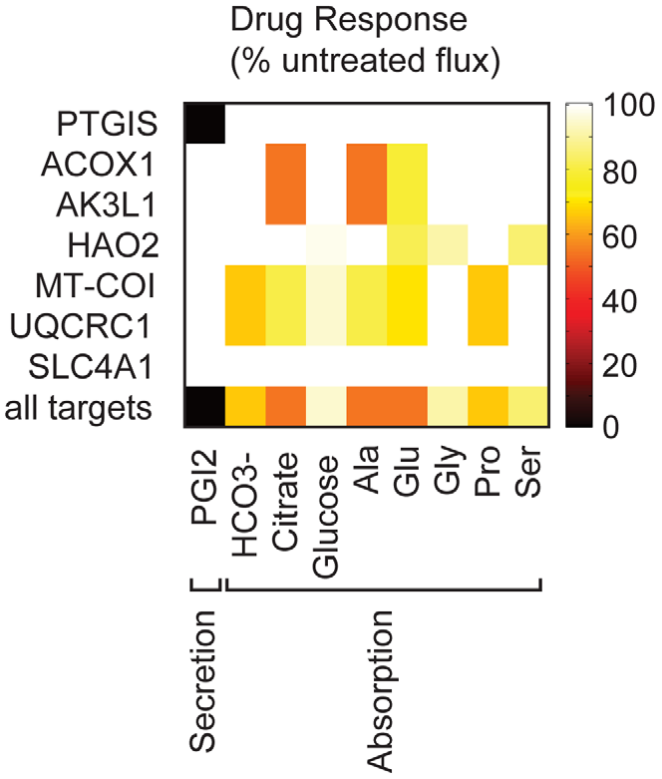
CETP inhibitor renal response phenotypes. Elements of the color matrix represent the percent of the maximum normal, untreated renal objective flux achievable by the CETP-inhibitor-treated normal kidney model. The x-axis corresponds to individual renal objective functions, and the y-axis corresponds to the predicted drug off-targets. Metabolite abbreviations are defined in Table 1. Only the subset of renal objective functions for which a drug response phenotype was predicted is displayed.

The integrative framework adopted for predicting causal drug targets associated with response phenotypes employed both structural bioinformatics tools as well as modeling techniques of systems biology (see Materials and Methods and Figure 4). The workflow begins with screening of the entire human structural proteome, with each subsequent step in the process narrowing the list of proteins ultimately into a set of targets for which a response phenotype was predicted upon functional inhibition. The first step of this process identified putative off-target drug binding sites using a ligand-binding site structural alignment algorithm (see Materials and Methods). The 41 predicted metabolic protein off-targets were the focus of this study (see Table S3), 28 of which had predicted drug binding sites overlapping with their functional sites. Simulated inhibition of these targets in the reduced kidney model (see Materials and Methods) predicted response phenotypes for 6 of the off-target proteins with respect to renal function (Figure 5). The results of all analysis steps for these 6 off-targets are summarized in Table 2. The expression of all of these targets was determined to be the most limiting for their associated metabolic reactions included in the reduced kidney model (see Materials and Methods), providing additional evidence supporting that inhibition of these targets would be expected to have at least some deleterious impact on those reactions.

**Table 2:** Drug side effect causal off-targets.

Official Symbol	PDB ID	Gene ID	SMAP Prediction	Functional Site Overlap	Reduced Model Reactions Limited by Expression	Impacts Renal Function in Simulation	Stronger Drug Binding Affinity	Cryptic Genetic Risk Factors	References Supporting Causal Drug Target Prediction
PTGIS	2IAG	5740	×	×	×	×	×		[26], [27]
ACOX1	1IS2^a^	51	×	×	×	×	×		
AK3L1	2BBW	205	×	×	×	×			
HAO2	1LTD^a^	51179	×	×	×	×		SLC3A1;SLC7A9;SLC7A10;ABCC1	[28], [29]
MT-COI	1V54^a^	4512	×	×	×	×		CYP27B1;ABCC1	
UQCRC1	1PP9^a^	7384	×	×	×	×		CYP27B1;ABCC1	

a Non-human protein structures were mapped to human genes via bi-directional BLAST against the human proteome and choosing the top hit only if it had E-value <10^−50^.

The renal response phenotypes for inhibition of two of the predicted drug off-targets were supported by existing scientific literature. Simulated PTGIS inhibition completely precluded PGI2 secretion. Based on the relation of renal PGI2 secretion to blood pressure (see Table 1), this inhibition would be expected to have a hypertensive effect. Experimental studies confirmed that PTGIS is associated with essential hypertension in humans [26] and that transgenic rats highly expressing human PTGIS exhibited decreased mean pulmonary arterial pressure despite treatment with monocrotaline to induce hypertension [27]. Inhibition of hydroxyacid oxidase 2 (HAO2) in the reduced kidney model led to reduced glutamate, glycine, and serine reabsorption suggesting a possible role for HAO2 in the hypertensive side effect following CETP inhibitor treatment based on the association of amino acid reabsorption with vasodilation and hypertension (see Table 1). HAO2 is highly expressed in human kidney [28] and was identified as a candidate quantitative trait locus for blood pressure in rat kidney in a study comparing normal to hypertensive rats [29].

**Figure 6:**
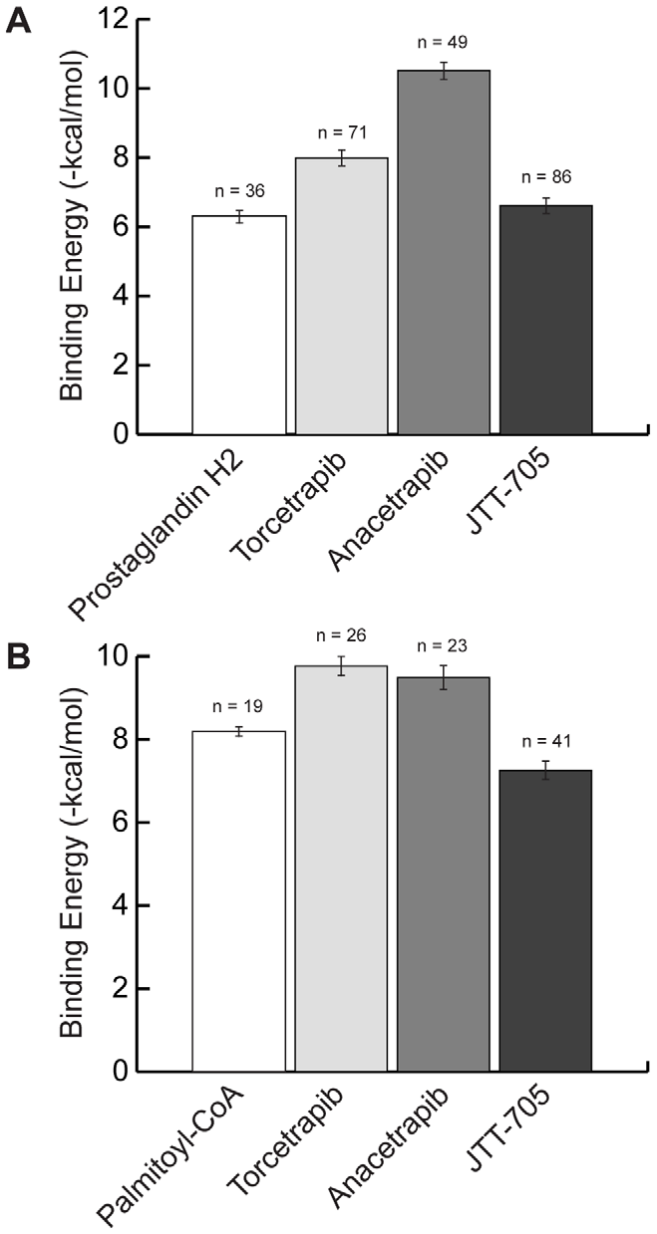
Differential causal off-target ligand and drug binding affinities. (**A**) Binding affinities of the prostaglandin I2 (prostacyclin) synthase protein for CETP inhibitors and prostaglandin H2, the endogenous substrate. (**B**) Binding affinities of the acyl-Coenzyme A oxidase 1, palmitoyl protein for CETP inhibitors and palmitoyl-CoA, the endogenous substrate. Each bar shows the mean binding energy predicted from docking trials. The standard error is indicated for each bar along with the number of predicted binding poses.

Two predicted causal CETP inhibitor off-targets, PTGIS and ACOX1, exhibited notable binding affinity differences when comparing docking results for their endogenous substrates to those for the three CETP inhibitors (Figure 6). The mean predicted binding affinity of PTGIS for its endogenous substrate prostaglandin H2 was weaker than for all three CETP inhibitors (Figure 6A). Anacetrapib was predicted to have the strongest mean binding affinity of all four tested molecules for PTGIS and JTT-705 the weakest of the three drugs. The predicted mean binding affinity of ACOX1 for its endogenous substrate palmitoyl-CoA was weaker than for torcetrapib and anacetrapib but stronger than the affinity of the protein for JTT-705 (Figure 6B). These results supported potential competitive inhibition of PTGIS and ACOX1 by torcetrapib and anacetrapib, but the predictions suggested a lesser effect of JTT-705 on ACOX1.

### Renal Disorders and Drug Treatment

Similar to the use of the model to test inhibitory effects on drug targets, the model was also used to predict genetic deficiencies that lead to renal disorders and drug off-targets that act synergistically with genetic deficiencies. Simulated gene knockouts predicted to impact renal objective functions are displayed in Figure S1, Figure S2 and Table S4. The 118 deficient genes predicted to cause disorders impacted a variety of renal secretions and absorptions to varying degrees. Thirteen of these deficiencies predicted total loss of at least one renal function (see Figure S2).

Some renal disorders were only predicted in the gene-deficient models in combination with drug treatment, not in the untreated gene-deficient models or in the normal drug-treated model, and are referred to in this study as cryptic genetic risk factors. Five such gene deficiencies were predicted (see Table S4). A deficiency in CYP27B1, which impacted vitamin D secretion alone, also exhibited defects in proline reabsorption when combined with drug treatment in simulation. Defects in three amino acid transport proteins (SLC7A10, SLC3A1, and SLC7A9) were predicted to decrease renal glycine reabsorption in combination with drug treatment along with the disorders predicted in the absence of drug treatment. The model deficient in the ATP-binding cassette sub-family C member 1 gene (ABCC1) was predicted to exhibit a cryptic deficiency in renal phosphate reabsorption under drug treatment. These predictions are of special importance because they suggest that these renal phenotypes would only surface in gene-deficient individuals under certain conditions, such as when treated with CETP inhibitors.

### Model Evaluation and Validation

Multiple evaluations were performed to analyze and validate the content of the reduced kidney model. The reduced kidney model effectively predicted activity of significantly expressed metabolic genes. The ability of our modeling approach to correctly and robustly predict activity of highly expressing genes was evaluated by a five-fold cross validation (see Materials and Methods). Our approach showed significant recall of the 20% most highly expressed metabolic genes, p-value  = 4.57×10^−22^. This observation is especially notable since the reduced kidney model was not a global model of kidney metabolism, and the result suggests the relative importance of the renal functions captured by our model within the context of total kidney gene activity.

**Figure 7:**
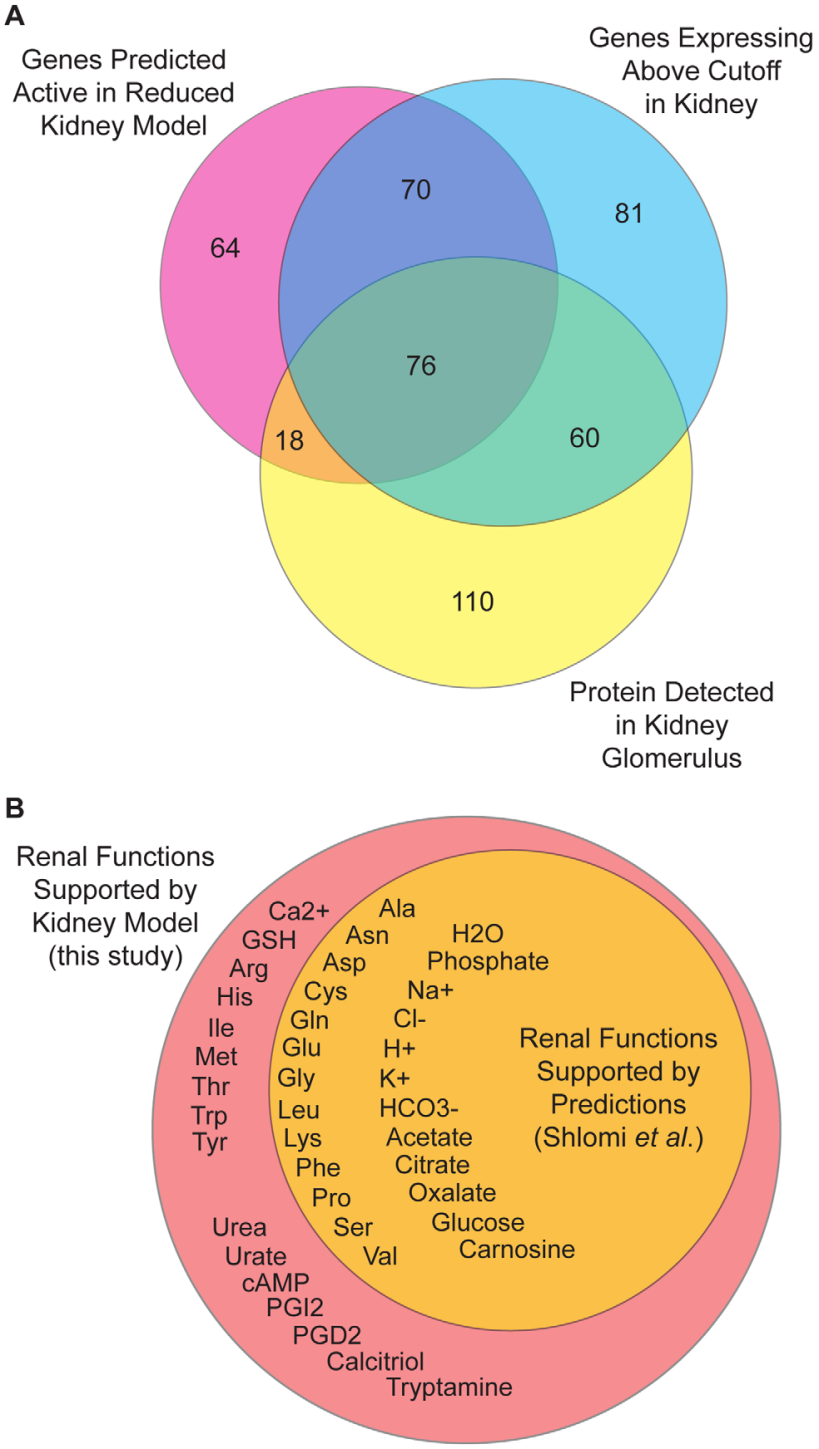
Comparative reduced kidney model evaluation. (**A**) Overlap of gene activity predictions with genes expressing above the significance threshold. Regions of the diagram are approximately proportional to their associated set sizes. The magenta circle represents the set of genes predicted active in the reduced kidney model. The cyan circle represents the set of Recon1-associated genes with expression levels above the significance threshold in the kidney tissue data. The yellow circle represents the set of genes encoding proteins that were detected in normal human kidney glomerulus tissue. (**B**) Renal metabolic objectives supported by predicted reaction flux states. The orange circle represents renal metabolic objectives supported both by the kidney model developed in this study and a kidney model derived from the reaction activity predictions of Shlomi *et al*. The red circle represents renal metabolic objectives supported only by the kidney model from this study. Metabolite abbreviations are defined in Table 1.

We compared the metabolic gene activity predictions from the reduced kidney model to the set of significantly expressed genes as well as to a proteomic dataset derived from normal, healthy human kidney glomerulus tissue [30] (Figure 7A). A total of 164 genes active in the reduced kidney model, 72% of the predicted activities, were supported by either significantly expressed mRNA levels, high-confidence protein detection, or both (see Table S1 for a detailed list). The remaining 64 gene activities accounted for in the model include 23 genes with no corresponding microarray probesets, and therefore not experimentally measured mRNA, and 41 genes that were determined to express more marginally below the established significance threshold. Despite a strong overlap between the transcriptomic and proteomic datasets, there were also large proportions of both which are unique. This disagreement may be due to tissue samples being taken from different kidney sub-tissues in each experiment, absent probesets on the microarray, or the propensity of mass spectrometry proteomic experiments to produce false negatives. All of the counted activities in Figure 7A were included in the full human metabolic network, signifying that the reduced kidney model was not a global kidney model and that there is potential for expansion to account for more metabolic functions than those of concern in this study.

The literature-curated renal functions achievable by the kidney model were also compared to those achievable by a model derived from the predictions of Shlomi *et al* (Figure 7B). While the kidney model developed in this study was compatible with all 41 curated renal functions, the predictions of Shlomi *et al* were only compatible with 25 functions. This difference in functionality was due to false negative inactivity predictions made by Shlomi *et al* such as inactive urea transport, prostaglandin synthesis, and ATP synthesis. These results underscore the need to manually curate automatically generated metabolic network reconstructions and the advantage of integrating objective functions with context-specific modeling.

Next, the model was functionally validated by comparing the gene deficiencies predicted to cause renal disorder to disease phenotypes in the OMIM database collected from clinical studies. Twenty known gene deficiencies leading to specific disease phenotypes were accurately predicted using the model (see Table S4). Loss of function mutations in the gene encoding 25-hydroxyvitamin D3-1-alpha hydroxylase (CYP27B1) have been linked to vitamin D-dependent rickets type I in both human patients [31] and pigs [32] consistent with the predicted inability of the gene-deficient model to secrete calcitriol. Hypouricosuria, low urinary excretion of urate, is a symptom of xanthinuria that is caused by xanthine dehydrogenase (XDH) deficiency [33], which is consistent with the deficient model's inability to excrete urate. Similarly, hypouricemia, low blood serum urate, is a consequence of nucleoside phosphorylase (NP) deficiency [34] also predicted in the model. Deficiency of aromatic L-amino acid decarboxylase (DDC) leads to increased urinary excretion of 5-hydroxytryptophan [35], which is consistent with the decreased ability to reabsorb tryptophan and secrete tryptamine predicted through simulation. Mutations in the mitochondrial cytochrome c oxidase gene (COX6B1) lead to de Toni-Fanconi-Debre renal syndrome, whose symptoms include a deficiency in the renal reabsorption of glucose, amino acids, and bicarbonate [36], [37], all of which were predicted in the model. Deficiencies in seven NADH dehydrogenase genes all lead to hypoglycemia, confirmed in simulation, and a decreased ability to oxidize citrate and glutamate [38], reactions important for indirect renal reabsorption of citrate and glutamate in the model. Proline dehydrogenase (PRODH) deficiency causes an inability to oxidize proline in kidney and other tissues leading to hyperprolinemia that includes increased urinary excretion of proline as a symptom [39]–[41], which is also consistent with the predicted decrease in renal proline reabsorption. Deficiencies in two genes that take part in the ubiquinol-cytochrome c reductase complex III (UQCRQ and UQCRB) lead to proximal tubulopathy, including an inability to reabsorb amino acids [42]; the gene-deficient model exhibited reduced renal reabsorption of alanine, glutamate, and proline. Fumarate hydratase (FH) deficiency leads to defects in glutamate oxidation in kidney and other tissues [43], [44], which is also consistent with the decreased indirect renal reabsorption of glutamate predicted by the model. Renal glucosuria, recapitulated in the model, results from deficiency in a sodium-glucose transporter (SLC5A2) [45]. Dicarboxylicamino aciduria [46] exhibits impaired renal glutamate and aspartate reabsorption and hypoglycemia resulting from a deficient glutamate transporter (SLC1A1), all symptoms predicted by the model. Severe dehydration is one symptom resulting from another deficient transporter (SLC5A1) [47], confirmed through decreased reabsorption of water in the model. These results qualitatively describe the ability of our modeling approach to predict perturbed phenotypic states.

To more rigorously quantify the predictive ability of our model simulation approach, we performed area under receiver operating characteristic (AROC) analysis based on not only the abovementioned clinical validations of our gene-deficient phenotype predictions but based on the entire set of such known clinical phenotypes that could potentially have been investigated using our model (see Figure S3 and Materials and Methods). The sharp declines in rates with increasingly stringent classifier ratio thresholds (see Figure S3) reflect the likely low coverage of actual disorder phenotypes by existing clinical studies. Nevertheless, our approach performed very well based on this analysis, with an AROC of 0.7565. Permutation trials resulted in a mean AROC of 0.5112, in close agreement with the expected theoretical randomly achievable AROC of 0.5. Our approach achieved a significantly greater AROC than could be expected by chance, p-value  = 8.71×10^−70^. Given the relatively low number of actual clinical negatives available (see Table S5), we also assessed the significance of our prediction results based purely on the true positive rates determined through the AROC analysis. The mean true positive rate of our results in this analysis was 0.2859, significantly greater than the 0.0215 mean true positive rate obtained randomly, p-value  = 3.29×10^−127^. These analysis results illustrate that our approach for predicting perturbation phenotypes exhibits both favorable sensitivity and specificity based on actual clinical data and should hold not only for predicting genetic deficiency phenotypes but also enzyme inhibition by drugs, which exhibits a similarly deleterious phenotypic effect.

### Parameter Sensitivity Analysis

In order to assess the effects of some of the critical assumptions made in the model development and simulation procedures, we performed sensitivity analysis with respect to the predicted renal disorder phenotypes.

**Figure 8:**
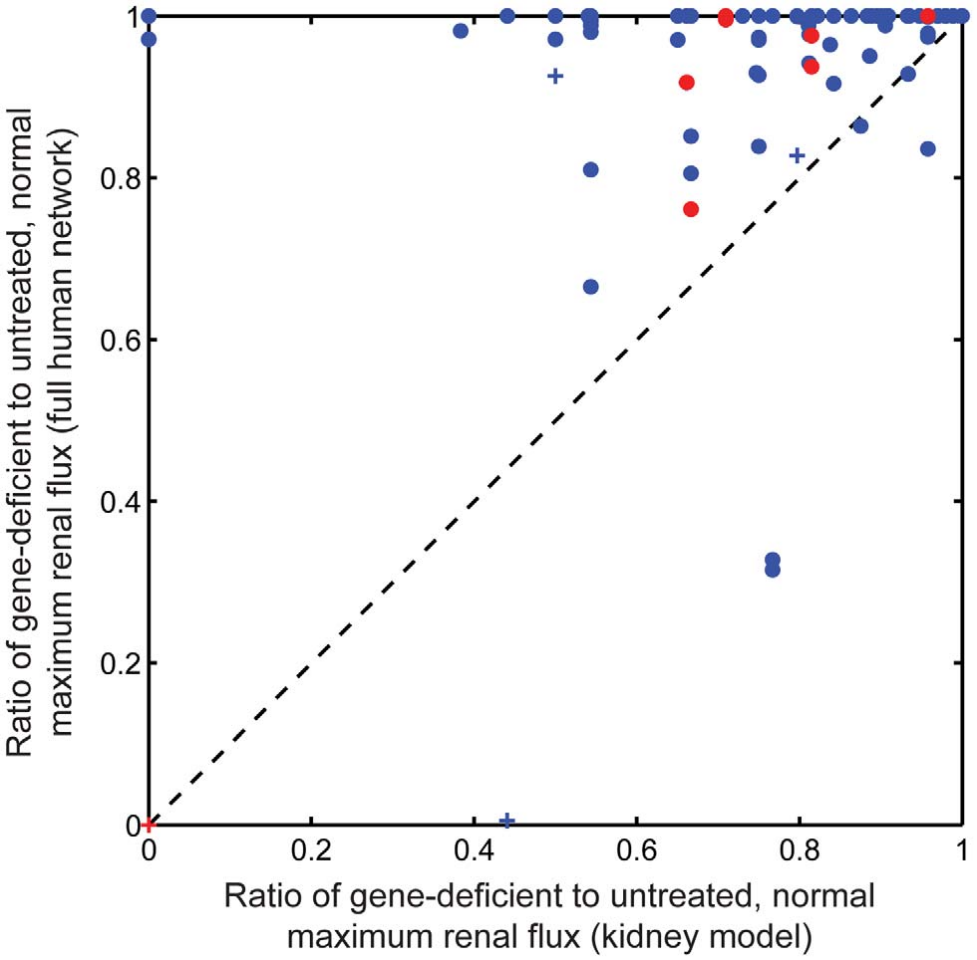
Predictive ability gained by modeling. The dotted black line is the line y = x for ease of visual comparison. Red marks represent predictions resulting from inhibition of a predicted CETP inhibitor off-target. Blue marks represent predictions resulting from non-drug-target gene inhibition. Pluses represent predictions validated in the OMIM database. There are 608 marks in total plotted and exact and partial overlap of some marks precludes complete visual resolution.

First, we compared the predictive capability of our reduced kidney model to that of the original, unconstrained human Recon1 metabolic network. The same approach to simulating renal disorder states was employed using both models (see Materials and Methods). We simulated all single gene knock outs in both models and assessed the renal disorder phenotypes with respect to each individual component of the renal objective function based on the ratio of maximum objective flux in the perturbed state to maximum objective flux in the unperturbed state. Comparing the results achieved by each model (Figure 8), it is apparent that although there are a few cases where both models predict an equal degree of renal disorder given the same genetic perturbation, the vast majority of disorder phenotypes are more apparent in the reduced kidney model than in Recon1 alone. In fact, 427 out of the 608 (71%) disorder phenotypes predicted by the reduced kidney model showed no degree of disorder relative to the unperturbed state in Recon1, including 36 of the most severe phenotypes for which a total loss of renal function was predicted by the reduced kidney model. These observations display the predictive ability gained through integration of the gene expression data via the GIMME algorithm, incorporating metabolomics data to set exchange constraints, and the addition of six key membrane transport reactions during the limited function-enabling manual curation of the model. These reactions involve the transport of prostaglandins I2 and H2, calcitriol, and carnosine. It should be noted that the 7 disorders for which Recon1 predicted a more severe phenotype than the kidney model result directly from the addition of these transporters in that these transporters have enabled additional pathways in the kidney model that are absent in Recon1. All but one of the predictions concerning CETP inhibitors showed a clearer phenotype in the kidney model as well; this off-target is PTGIS for which both models predict a complete loss of function when fully inhibited. Finally, 28 out of the 33 clinically validated phenotypes are predicted more noticeably by the kidney model, 17 of these showing no disorder phenotype in Recon1. Overall, this comparison establishes the relative contribution of context-specific modeling in studying disorder and drug response phenotypes.

Second, we investigated the sensitivity of our drug off-target response phenotype predictions to the variability of two important parameters used in our simulations, the system boundary flux constraint, set as equal fractions of the upper bound on renal objective fluxes (see Materials and Methods), and the degree of enzymatic activity inhibition assumed to result from drug treatment.

The system boundary flux constraint was imposed upon demand and exchange reactions other than those optimized during a given simulation. By default we set this constraint assuming that all allowed boundary fluxes can carry an equal fraction of the potential maximum renal objective flux. This assumption was made to allow all pathways that could possibly contribute to the objective to be used simultaneously in the optimal flux state, providing the most flexible state while maintaining maximum sensitivity of our model to additional system perturbations such as gene deficiencies or drug effects. This approach was unbiased in that it did not favor any possible pathway over another in achieving a set objective without imposing additional constraints, which may not always reflect biological reality but was the most conservative assumption in the absence of additional experimental data required to more precisely set these flux constraints. In our sensitivity analysis, we varied this parameter between 0 and 1000 flux units, the absolute lower and upper magnitudes possible in our model, and repeated the simulations of drug off-target effects. The result of this analysis (Figure S4) was captured in the normalized sensitivity coefficient computed for each simulation (see Materials and Methods). The coefficient can vary between negative and positive unity and displays the deviation from a base result, the primary predictions we have presented in this study. The base result is indicated by a black star in Figure S4, and the parameter value in this case equals 13.5 flux units.

It is clear from Figure S4 that PTGIS inhibition resulted in the same renal disorder phenotype regardless of the value of the system boundary flux constraint parameter. This was because there was only one pathway in the model by which prostaglandin I2 could be secreted. Most other disorder phenotype predictions begin to diverge from the base result around a parameter value of 200 flux units, a fairly permissive value, which shows that the predictions were fairly robust to variability of this parameter. The closer to 1000 flux units this parameter was set, the more completely alternative pathways could compensate for a loss of function in the simulations. If alternative pathways existed to achieve a renal function, it was guaranteed that the ability to predict a disorder phenotype with respect to that function would be completely lost at the maximum possible parameter value of 1000.

We similarly analyzed the sensitivity of our predictions to changes in the degree of enzyme inhibition assumed to follow from drug treatment (Figure S5). For the primary results presented in this study, we assumed complete inhibition of activity by the drug, corresponding to a fraction of maximum enzymatic reaction flux equal to 0 in Figure S5. Similar to the default setting of our system boundary flux constraint, this default of complete inhibition was chosen in order to maximize the sensitivity of our model in detecting disorder phenotypes. Most of the phenotypes were still detectable to varying degrees with as much as 25% of the maximum activity of drug targets. The predicted phenotypes associated with PTGIS, ACOX1, and AK3L1 were especially robust to variation in degree of inhibition, still exhibiting a phenotype near 50% of maximum activity. Decreased glucose and bicarbonate reabsorption under drug-induced MT-COI and UQCRC1 inhibition exhibited the most sensitivity to variability in this parameter, although none of the predicted phenotypes required complete inhibition of the drug target in order to be detected.

## Discussion

A novel approach for making functional predictions of drug response phenotypes has been introduced that integrates techniques of both structural bioinformatics and systems biology. Although the current study focused on a specific metabolic system, the general methodology excluding techniques particular to metabolic modeling are extensible to other systems such as signaling or transcriptional regulation. Non-metabolic protein drug off-targets are predictable using the same structural analysis tools, and many such off-targets have indeed been predicted as well for CETP inhibitors [4].

The context-specific organ metabolic modeling strategy employed in this study represents an improvement upon previous efforts in this realm. Model development algorithms such as GIMME [13] or that developed by Shlomi *et al*, when integrated with multiple omics datasets, can lead to more biologically realistic models. It is also of critical importance to include context-specific metabolic objective functions in the model development process in order to yield a fully functional and predictive model, as is evident from the functional comparisons of models performed in this study.

As an early effort at modeling such a context-specific metabolic system it is important to discuss the limitations of our model. Although the functional validations presented here are compelling, currently available clinical data only permits the assessment of a subset of the predictions possible in the model. Also, the functional portion of the model, the reduced kidney model, does not and is not intended to represent a global model of kidney metabolism but only the specific renal functions studied in this work. As such, our model does not fully resolve of complexity of the human kidney. The human kidney fulfills a number of functions not studied here and is a spatially distributed system across multiple distinct tissue types. Here we have summarily replaced the various kidney sub-tissues with a single, net system model. Because we integrated expression data with curated renal functions that operate across multiple kidney tissues, it is likely that our model approximates a superset of the metabolic pathways supporting these functions. Although we have made several simplifying assumptions in the model development process, even the current level of model validation suggests that the gene and reaction content of the model is fairly accurate and that simulations in this model indeed hold predictive capability.

The simulation approach taken, optimization of a linear objective function, does not fully capture the full physiological role of the kidney. The goal of these simulations was to determine drug-target effects that may limit the capacity of the kidney to move towards a homeostatic nominal state from a state of high blood pressure, thereby decreasing the capacity of the kidney to lower blood pressure. This strategy is appropriate for the goals of the current study but would not be appropriate to simulate all physiological states of interest in the kidney. On a related note, the choice to define a disorder state based on the ratio of perturbed to unperturbed maximum achievable renal objective flux demonstrates a difference in the capacity of the renal function and not necessarily a precise flux state. Therefore this strategy too is not appropriate for modeling all physiological states.

The predictions made for CETP inhibitors in this study serve as illustrative examples of many important implications that this approach has for drug development and personalized medicine. Predicted causal off-targets for renal metabolic disorders related to blood pressure may be responsible in part or full for the clinically observed hypertensive side effect of torcetrapib. The evidence resulting from this study suggests that PTGIS and ACOX1 are both potential causal torcetrapib off-targets, the inhibition of which may explain the side effect of hypertension. In addition, AK3L1, HAO2, MT-COI, and UQCRC1 may also play a role in this side effect as we have predicted, although our docking trials did not suggest that they are bound as strongly by torcetrapib. The specific predicted deficiencies in renal function associated with the drug off-targets can serve as biomarkers to be measured in patients participating in clinical trials. A positive correlation of these biomarkers with side effects would lend support to the predictions of this study and confirm these biomarkers as risk indicators in future patient treatment. It is important to note that although these predictions comprise the basis for a renal filtration and secretion-based hypothesis explaining the hypertensive side effect of torcetrapib, these results do not refute the hypothesis based on a RAAS-mediated mechanism. These two hypotheses are not mutually exclusive and could potentially contribute alternatively or synergistically to the clinically observed side effects. This possibility illustrates the major tenet for systems biology: studying a single protein or even a single pathway is not necessarily sufficient to explain complex biological phenomena.

Aside from the confirmation that some of our predicted off-targets are known to be involved in renal disorders, we do not currently present direct experimental verification that torcetrapib binds and inhibits the predicted targets and that this inhibition leads to the predicted response phenotypes. Although this would be the obvious next step, a retrospective validation is currently hampered by the availability of the drug and the nature of the phenotypes both predicted and known. Ideally, relevant physiological studies would be carried out during actual clinical trials, when a method such as ours would be most useful, in preclinical and clinical phases of drug development.

The extended structural analysis of causal drug off-targets to identify differential binding affinities for endogenous substrates and drug molecules suggests possible differences in drug response phenotypes across the CETP inhibitors tested. The results suggest that anacetrapib may potentially lead to a similar response phenotype to that of torcetrapib, while JTT-705 may not carry the same adverse effect, at least with respect to the off-targets detailed in this study. This particular type of analysis may aid in differentiating between likely response phenotypes expected for chemically and functionally similar drugs. Results of the computational pipeline for interaction prediction between proteins and CETP inhibitors employed in this study, SMAP and docking, have yet to be confirmed experimentally. Although we are currently unable to provide direct experimental evidence for the off-target interaction predictions for this class of drugs, multiple recent studies have shown experimental support for the general efficacy of this approach for interaction prediction [48], [49].

The predicted renal metabolic disorders with a genetic basis suggest classes of individuals in which treatment with CETP inhibitors may pose a higher risk for adverse side effects. These predictions suggest a likely relationship between participants in torcetrapib clinical trials exhibiting symptoms of these disorders and the observed adverse side effects. The concept of cryptic genetic risk factors for drug treatment introduced in this study suggests a novel approach to personalized medicine. Should polymorphisms within these genes be clinically linked to side effects of drug treatment, the result would comprise a basis for genetic screening to assess the risk of drug treatment for future patients. Given that these cryptic risk factors are not expected to elicit the predicted abnormal phenotypes in the absence of drug treatment, identification of causal polymorphisms through association studies could only occur during clinical phase when a sufficient number of patients could be observed to gain the statistical power needed to draw significant correlations.

As illustrated above, this approach for *in silico* drug testing could become an indispensible tool during the pre-clinical and clinical phases of new drug development for studying the nature of adverse side effects. In addition, this platform holds obvious potential for analyzing drug efficacy in general and identification of potential beneficent drug side effects that may be useful for drug repositioning and could also be easily adapted for studying combinatorial drug treatment. For a failed drug like torcetrapib, results from this approach could reinitiate the drug development process, providing new insight to help target patients who could benefit from the treatment without the risk of serious adverse side effects.

## Materials and Methods

### Prediction of CETP Inhibitor Drug Off-Targets

The binding site for CETP inhibitors on the CETP structure and the predicted off-target binding sites for this class of drug across the proteome were assumed to be as previously predicted using the SMAP program [4], which implements the Sequence Order Independent Profile-Profile Alignment (SOIPPA) algorithm to identify significant structural similarity to a given ligand-binding site [3]. The results contained proteins from all organisms represented in the PDB, not just human structures.

### Mapping Off-Target Proteins to the Metabolic Network

In order to integrate the result of drug off-target predictions with the metabolic network, it was necessary to first map all PDB structures (http://www.pdb.org) corresponding to human metabolic proteins included in Recon1, downloaded from the BiGG database, to their respective gene identifiers as represented in Recon1. The BiGG database requires registration and a password, which can be requested by visiting (http://bigg.ucsd.edu/bigg/home.pl). The UniProt ID mapping tool (http://www.uniprot.org/) was used to map PDB structures corresponding to human proteins to gene identifiers linked to metabolic reactions in Recon1 accounting for all predicted human metabolic protein drug off-targets. All non-human predicted metabolic protein drug off-targets were mapped to their human orthologs using the Basic Local Alignment Search Tool (BLAST) [50] to perform a bi-directional BLAST with a mutual best hit criterion. BLAST was also used to resolve inconsistencies in functional annotation between Recon1 gene-protein-reaction associations (GPRs) and gene annotations from the Entrez Gene database (http://www.ncbi.nlm.nih.gov/sites/entrez?db=gene) with respect to predicted drug targets, leading to the reannotation of three Recon 1 GPRs. The overall result of this mapping was that 97 metabolic reactions in Recon1 were linked to 41 predicted CETP inhibitor off-targets.

### Enzyme Inhibition Analysis

The metabolic enzymes predicted as CETP inhibitor off-targets using SMAP were evaluated to determine potential enzymatic inhibition by the drug. The predicted drug-binding sites of the putative off-targets were compared to endogenous ligand-binding sites from existing PDB protein-ligand complex structures (http://www.pdb.org) and catalytic sites from the Catalytic Site Atlas (http://www.ebi.ac.uk/thornton-srv/databases/CSA/). Ligand-binding sites were defined as amino acid residues lying within 4.5 Å from atoms of the ligand. Drug-binding sites were defined as residues aligned with the cholesteryl ester binding sites on the CETP structure using SMAP. Overlap between endogenous ligand-binding sites and drug-binding sites was defined by a sharing of any amino acid residues between the sites. The function of predicted drug targets present in Recon1 with at least a partial such overlap was considered to be competitively inhibitable by the drug.

### Protein-Ligand Docking

Enzyme substrates were identified from Recon1 reaction formulas. Certain molecules (H^+^, H_2_O, O_2_, phosphate, ferricytochrome C, and ferrocytochrome C) were excluded from docking trials due to size or structural challenges prohibiting a useful docking result for the purposes of binding affinity predictions. All protein structures used in this study were downloaded from the PDB (http://www.pdb.org). Three-dimensional structures for endogenous enzyme substrates were downloaded directly from the PDB if available. If the PDB ligand structure did not exist or was non-functional for docking, the structure was searched for in PubChem (http://pubchem.ncbi.nlm.nih.gov/). The subsequently downloaded SDF file was converted to PDB format using the ChemAxon web applet available at the PDB website (http://www.rcsb.org/pdb/ligand/chemAdvSearch.do). If the three-dimensional ligand structure could not be found in PubChem, the two-dimensional structure was derived from the canonical SMILES [51] representation of the compound available in PubChem and then converted to a three-dimensional structure in PDB format using the Clean3D Fine Build tool available through the Marvin web applet (http://www.chemaxon.com/marvin/sketch/index.jsp). The three-dimensional structures for glycolipids were derived from their KEGG glycan structures (http://www.genome.jp/kegg/glycan/) using SWEET-II (http://www.glycosciences.de/spec/sweet2/doc/index.php).

Protein structures were pre-processed for docking using AutoDockTools (ADT) version 1.5.2 [52] by adding polar hydrogen atoms, removing all non-protein molecules from the PDB structure including water, detergents, and ligands, adding Kollman charges to the protein and converting it to PDBQT format. Ligand structures were also prepared using ADT, using the default method for preparing ligands for docking that adds hydrogens and charges. The default rotatable bonds were accepted as well, and the structure was converted to PDBQT format. The search space for docking was determined visually by centering the Grid Box in ADT central to the experimentally determined binding site of the ligand and expanding the dimensions of the cubic search space to just completely encompass the site.

Docking was performed using AutoDock Vina [53] with default parameter settings other than the search space specification described above, and the mean predicted binding affinity from the set of predicted binding poses was accepted as the true binding affinity for each docking run. The predicted binding affinities for endogenous substrates were compared to the affinity of the same site for the CETP inhibitor drugs in order to make predictions about differential responses with respect to each of the drugs.

### Renal Objective Function

As the preliminary step in modeling human renal function, the scientific literature was reviewed to compile a list of specific metabolic functions of the kidney, with a focus on functions implicated as determinants of blood pressure. This list includes a number of renal reabsorptions and secretions. Each function in this list was tested for compatibility with Recon1, downloaded from the BiGG database (http://bigg.ucsd.edu/bigg/home.pl), by performing flux balance analysis (FBA) on the fully unconstrained network optimizing for the given function. Those functions compatible with Recon1 were those that could achieve a positive flux and are summarized in Table 1. These metabolic functions were combined with a basic ATP maintenance function to form a single model reaction that represents the kidney's ability to filter the metabolic content of blood with preference for lowering blood pressure. This model reaction was used as the objective function in developing the metabolic kidney model and is referred to as the renal objective function in this study. All stoichiometric coefficients in this reaction were set equal to one, which is a safe assumption for the model development step as this only significantly impacts the magnitude of fluxes through pathways that support each individual renal objective and not generally whether or not certain fluxes will be active in the resulting model. For the full renal objective function reaction to be seen as useful in performing simulations, more careful balancing of these coefficients based on experimental evidence would be required. As such, the full renal objective function was not used in any subsequent simulations with the model, instead being substituted as an objective by the reactions representing individual reabsorptions or secretions.

Metabolite exchange and transport reactions needed to achieve some of the renal functions were also added to the network. It was observed that Recon1 as a base model could not achieve flux through certain key renal metabolite reabsorptions: sodium, calcium, chloride, potassium, and oxalate. These deficiencies were corrected for by simply adding demand fluxes for these metabolites in the cytosol model compartment. Demand fluxes were also added for the remaining kidney reabsorptions and secretions as well to enable an array of simulations involving individual components of the renal objective function to be tested. In the case of reabsorption, this allows for direct reabsorption of metabolites in addition to indirect reabsorption in which the absorbed metabolite is first metabolized into other compounds and then reabsorbed into the blood, as is the primary mechanism of reabsorption for some metabolites, such as reduced glutathione (GSH) [54].

### Kidney Metabolite Exchange Flux Constraints

A preliminary model was created by imposing kidney-specific exchange flux constraints representing the metabolic exchanges the kidney carries out with the blood and urine. The preliminary model was initialized by loading Recon1 into the COBRA Toolbox [55] and, by default, unbounding all reaction fluxes by setting them to the default maximum magnitude of 1000 flux units. Next, the renal objective function was added to the network as a single reaction. Exchange fluxes for kidney secretion objectives were constrained to preclude uptake of those metabolites to achieve the renal objective, forcing the model to synthesize those metabolites in order to secrete them. The resulting preliminary model included 407 exchange fluxes, only 49 of which were explicitly unconstrained based on literature-curated kidney functions and the most basic of metabolic precursor requirements. The basic metabolic exchanges assumed to take place include ions and other inorganic compounds.

The Human Metabolomics Database (HMDB) (http://www.hmdb.ca/) was queried to derive further evidence in support of allowable exchange fluxes for the kidney. All 407 exchange metabolites in the preliminary model were searched in HMDB for experimental detection in specific biofluids and tissues. Those metabolites detected both in the blood and kidney tissue were assumed to be freely exchangeable in the kidney model, leading to 78 more explicitly unconstrained exchanges beyond what was derived from basic and curated kidney-specific metabolic functions. This assumption is based on the kidney's physiological role of filtering the blood and the observation that if both the blood and kidney contain a metabolite, it must either be exchanged between the two or synthesized separately in both. In the former case, this data provides evidence of that exchange. In the later case, although the model might allow a biologically unrealistic exchange, because the metabolite exists in both blood and kidney, the impact on simulations using the resulting model should be merely quantitative in terms of the maximum renal objective fluxes achievable by the unperturbed model. The integration of gene expression data in the model development process described below should reduce the propensity for biologically unsound metabolic pathway activation that could follow from precursors introduced by any biologically unsound exchanges. Those metabolites detected both in the urine and kidney were assumed to be possible excretions, and exchange constraints were set accordingly. Excretions determined utilizing the urine metabolomics data mostly showed redundancy in determining exchange constraints with exchanges determined using blood data or literature curation with the exception of 4 additional metabolites. The remaining 276 exchange fluxes for which no evidence was found to support were tentatively constrained to 0 flux units.

The resulting preliminary model was again tested for the ability to achieve all kidney-specific metabolic functions. It was found that this model could not absorb and metabolize GSH, without also absorbing oxidized glutathione, the exchange of which was subsequently unconstrained. Also, L-threonine and L-methionine could not be absorbed and metabolized in this model without exchange of 2-hydroxybutyrate and 2-methylcitrate, the exchanges of which were similarly unconstrained as a corrective measure. The resulting preliminary model could still achieve all the same renal objectives as the fully unconstrained model. As a final preliminary constraining measure, all system effluxes were bound to equal fractions of the default upper bound on influxes of 1000 flux units; we term this parameter the system boundary flux constraint. This was done so that any available direct or indirect reabsorption pathways could possibly be used to achieve metabolite reabsorption without biasing the model towards use of any particular pathways without further evidence. This represents the state of the model just prior to final processing using the GIMME algorithm. The fitting of the allowable fluxes to the gene expression data by GIMME ultimately determined the usable reabsorption and secretion pathways in accordance with gene expression.

### Gene Expression Microarray Data Processing

Two gene expression microarray dataset for normal, healthy kidney tissue [56] were obtained from the GEO database (http://www.ncbi.nlm.nih.gov/geo/), accession GSE803. The two background-subtracted datasets were first normalized using a global normalization factor equal to the sum of probe intensities from the first dataset divided by the sum of probe intensities from the second dataset to account for any systematic differences in procedure between the two experiments. The resulting data were then normalized using the Lowess method [57] to reduce random noise. The resulting normalized datasets were then weighted equally as replicates in determining the final data for integration with the human metabolic network by taking the mean of the two normalized datasets.

The gene-protein-reaction associations (GPRs) in Recon1 use Entrez Gene IDs to annotate reactions in the network. To map the data from the AffyHG-U95 chips to Recon1, all genes included in Recon1 were mapped to corresponding AffyHG-U95 probesets using Bioconductor [58] and the most recent chip annotations [59]. A single expression value was then assigned to each gene in Recon1 based on the maximum normalized data value associated with any of the probesets mapped to a given gene. Next, a single expression value was assigned to each reaction in Recon1 by evaluating the Boolean rules in the GPRs with respect to the normalized expression data. The minimum data point was chosen for genes linked by an AND operator in a GPR, and the maximum data point was chosen for genes linked by an OR operator in a GPR.

Finally, a significant expression threshold was established for subsequent use in the GIMME algorithm. This was done by fitting the normalized gene expression data to a Gaussian distribution, estimating the mean and standard deviation of this distribution, and calculating p-values associated with each data point by subtracting the cumulative distribution function from one. The normalized data value corresponding to the p-value closest to but not exceeding 0.05 was chosen as the significance threshold; this resulted in a threshold of 991.3698 for the normalized expression data.

### Implementation of GIMME to Obtain Metabolic Kidney Model

To integrate the renal objective function and kidney gene expression data with the preliminary model to derive a functional kidney model, the GIMME algorithm [13] was implemented. The GIMME algorithm takes a metabolic network model, a gene expression dataset, and specified required metabolic functions as input and solves a linear programming optimization to yield the network flux activity state that maximizes the specified functions while remaining as consistent as possible with the gene expression data. The complete renal objective function, the combination of all functions presented in Table 1, was set as the metabolic objective with a minimum requirement of 90% of the maximum possible flux set as a parameter for GIMME in determining the final kidney model. The reaction expression threshold parameter was set as described above. GIMME was run with these parameters and the normalized expression data and preliminary model as inputs. The resulting reaction activity predictions were used to constrain metabolic reactions yielding the full kidney model. Subsequently, the connected sub-graph of this full kidney model, which includes all functioning reactions possible for achieving the renal objectives, was isolated and is this portion of the model we focused on for validation and simulation. We refer to this sub-model as the reduced kidney model (available in SBML format as Dataset S1).

### Validation of Kidney Model Content

Gene activity predictions made when deriving the metabolic kidney model were compared to the set of expressed genes with normalized expression values above the significance threshold described above. Activity predictions were also validated against a comprehensive proteomics dataset from normal human kidney glomerulus tissue [30] for overlap with network-associated proteins detected with high confidence, that is, identified through detection of two or more peptides.

To evaluate the modeling approach used in this study, a five-fold cross validation was performed in which the data corresponding to the most highly expressed 20% of network-associated genes were excluded before deriving the kidney model. The ability of each approach to correctly predict the activity of these most highly expressed 20% of genes was measured from the overlap of predictions with the highly expressed gene set assuming a hypergeometric distribution, and the resulting probability was Bonferroni-adjusted.

### Simulating Drug Target Effects and Renal Metabolic Disorders

All predicted metabolic protein drug off-targets were tested in the kidney model to assess the drug response phenotype caused by inhibitory effects in this system. Inhibition of metabolic proteins by the drug was modeled by constraining corresponding reactions catalyzed by drug targets to 0 flux units. Simulations of the consequences of these drug effects were performed using FBA as implemented in the COBRA Toolbox [55] in the MATLAB programming environment. Each drug target was evaluated with respect to its impact on each individual renal function to determine if target inhibition by the drug leads to a renal deficiency relative to the untreated normal kidney model. This was done by optimizing single exchange or demand fluxes at a time, representing reabsorptions and secretions respectively, out of the full set listed in Table 1. The cumulative effect of all predicted drug targets being simultaneously inhibited was also tested against each individual renal function. Renal secretion fluxes were maximized in simulation. Renal reabsorption fluxes were set as unbounded and then maximized while the remainder of allowable uptakes were constrained to equal fractions of the default maximum magnitude of 1000 flux units. The additional constraints were imposed for reabsorption simulations in order to allow the resulting network flux state to include concurrently active alternative optimal direct and indirect reabsorption pathways rather than having to identify alternative optimal pathways by performing multiple simulations.

Single gene deficiencies were also simulated in the kidney model to assess their effects on renal function and their potential as risk factors for treatment with CETP inhibitors. Each of the genes annotated to reactions in Recon1 was knocked-out of the kidney model and simulations were run using the gene-deficient kidney model both with and without drug treatment to assess effects on each individual renal reabsorption and secretion.

Drug response and metabolic disorder phenotypes were assessed by taking the ratio of maximum gene-deficient, untreated renal function flux to maximum normal, untreated renal function flux. A ratio of less than unity indicates a deleterious phenotype. Predicted metabolic disorder phenotypes were validated against previous clinical studies as represented in the Online Mendelian Inheritance in Man (OMIM) database (http://www.ncbi.nlm.nih.gov/omim/).

Cryptic genetic risk factors for drug treatment were also predicted for which the maximum gene-deficient, untreated renal objective flux equals the maximum normal, untreated renal objective flux but the ratio of maximum gene-deficient, drug-treated renal objective flux to maximum normal, drug-treated renal objective flux is less than unity.

### Parameter Sensitivity Analysis

Sensitivity of our prediction approach to variability in parameters was performed through repeated simulation in which we varied the parameter value across the full range of possible values. We investigated sensitivity with respect to each parameter independently. A normalized sensitivity coefficient was calculated as the result of each of these simulations. This coefficient was calculated by first taking the percent difference in the predicted outcome relative to a base case, our primary results, and then dividing it by the maximum possible percent difference.

### Area under Receiver Operating Characteristic (AROC) Analysis

Benchmark data was collected from the OMIM database (http://www.ncbi.nlm.nih.gov/omim/) by searching for all metabolic disorders related to renal reabsorptions or secretions that are associated with deficiencies in genes included in the reduced kidney model. The resulting list of disorders was manually curated using literature references to identify precisely which metabolic renal reabsorptions and secretions were impacted. These included not only those renal functions captured in Table 1, but also other renal exchanges. All resulting reabsorptions and secretions that can have corresponding non-zero fluxes under unperturbed conditions in the reduced kidney model were included in our benchmark data set (see Table S5). Every phenotype in the benchmark data was investigated through our model as described for simulating drug target effects and renal metabolic disorders, taking the ratio of perturbed to unperturbed flux capacities as a measure of phenotype, where a ratio of one signifies no disorder phenotype and a ratio of less than one signifies some degree of disorder. Next, the ratio threshold for classifying normal versus disorder phenotype was iteratively set to assess the sensitivity and specificity of our approach for predicting true and false positives across the full range from zero to one. Note that a threshold of one was used by default for the main results presented in this study. The true positive rate was plotted against the false positive rate (see Figure S3), the ROC curve, and the AROC was computed using the trapezoidal rule for approximating definite integrals. The statistical significance of our result was determined by comparison to 100 permutation trials in which all reaction flux ratios, perturbed to unperturbed, were randomly shuffled for each simulated gene deficiency and AROC-analyzed. The permutation trials exhibited true positive and false negative rates expected for random disorder phenotype classification (see Figure S3), and thus comprised an appropriate assessment of the predictive ability of our model simulation approach relative to chance. One-sample left-tailed student t-tests were performed using an alpha value of 0.05 to assess the statistical significance of the AROC and mean true positive rate achieved by our model simulation approach relative to the permutation results.

## Supporting Information

Dataset S1 Reduced kidney model.
(0.50 MB XML)

Figure S1 Genetic deficiencies causing renal metabolic disorders. Elements of the color matrix represent the percent of the maximum normal, untreated renal objective flux achievable by the drug-treated gene-deficient kidney model. The x-axis corresponds to individual renal objective functions, and the y-axis corresponds to the individual gene deficiencies represented by their official gene symbols. Metabolite abbreviations are defined in Table 1, and official gene symbols are defined in Table S4. Only the subset of renal objective functions for which a metabolic disorder was predicted is displayed.
(0.53 MB TIF)

Figure S2 Genetic deficiencies causing renal metabolic disorders (continued).
(0.48 MB TIF)

Figure S3 ROC curves for gene-deficient phenotype prediction. The blue line represents the analysis of the predictions of the model simulations presented in this study. The red lines represent the analysis of 100 different permutation trials. The dashed black line is the line y = x.
(0.11 MB TIF)

Figure S4 System boundary flux constraint sensitivity. Only those drug targets and renal functions are shown for which a deficient phenotype was predicted. The x-axis is in units of flux. The black star represents the base case which is presented as our primary result.
(0.77 MB TIF)

Figure S5 Degree of drug-induced inhibition sensitivity. Only those drug targets and renal functions are shown for which a deficient phenotype was predicted. The x-axis values correspond to the fraction of maximal enzymatic flux achievable in the untreated simulation, which represents the constraint placed on associated reactions for each simulation. The black star represents the base case which is presented as our primary result.
(0.93 MB TIF)

Table S1 Kidney gene activity predictions. A one indicates that the given gene satisfies the criterion corresponding to that column.
(0.15 MB XLS)

Table S2 Kidney reaction activity predictions. Reactions with at least one non-zero flux bound are predicted active in the kidney model. All other reactions are predicted inactive in the kidney model.
(0.74 MB XLS)

Table S3 Metabolic protein drug target predictions. All predicted metabolic protein targets for CETP inhibitors were subject to multiple levels of analysis to determine their possible causal role in adverse drug response phenotypes. An x indicates a positive result with respect to column labels representing each level of analysis. The proteins are sorted by the number of analyses suggesting a causal role.
(0.03 MB XLS)

Table S4 Summary and validation of gene deficiencies causing renal metabolic disorders. The complete list of gene deficiencies predicted to cause renal metabolic disorders is presented. Specific impacted renal secretions and reabsorptions are also displayed using metabolite abbreviations as represented in Recon1. Clinically observed phenotypes validating the association of these genetic deficiencies with predicted metabolic renal disorders found in the OMIM database and literature are noted. These validated predictions are listed first in the table, and the remaining predictions are sorted by the type of renal disorders caused by the gene deficiencies in simulation.
(0.05 MB XLS)

Table S5 Clinical gene-deficient renal disorder benchmark data. The set of clinically-observed gene-deficient renal metabolic disorder phenotypes collected from the OMIM database are presented that were used to perform AROC analysis. Clinical positives are observed disorder phenotypes with respect to specific metabolite reabsorptions or secretions associated with a gene deficiency, and clinical negatives are disorder phenotypes that were clinically confirmed not to occur in association with a gene deficiency.
(0.03 MB XLS)
