# Supplementary figures and images for: Drug Off-Target Effects Predicted Using Structural Analysis in the Context of a Metabolic Network Model
Source: PLoS Comput Biol. 2010 Sep 23;6(9):e1000938. doi: 10.1371/journal.pcbi.1000938 (PMC2950675; doi:10.1371/journal.pcbi.1000938)

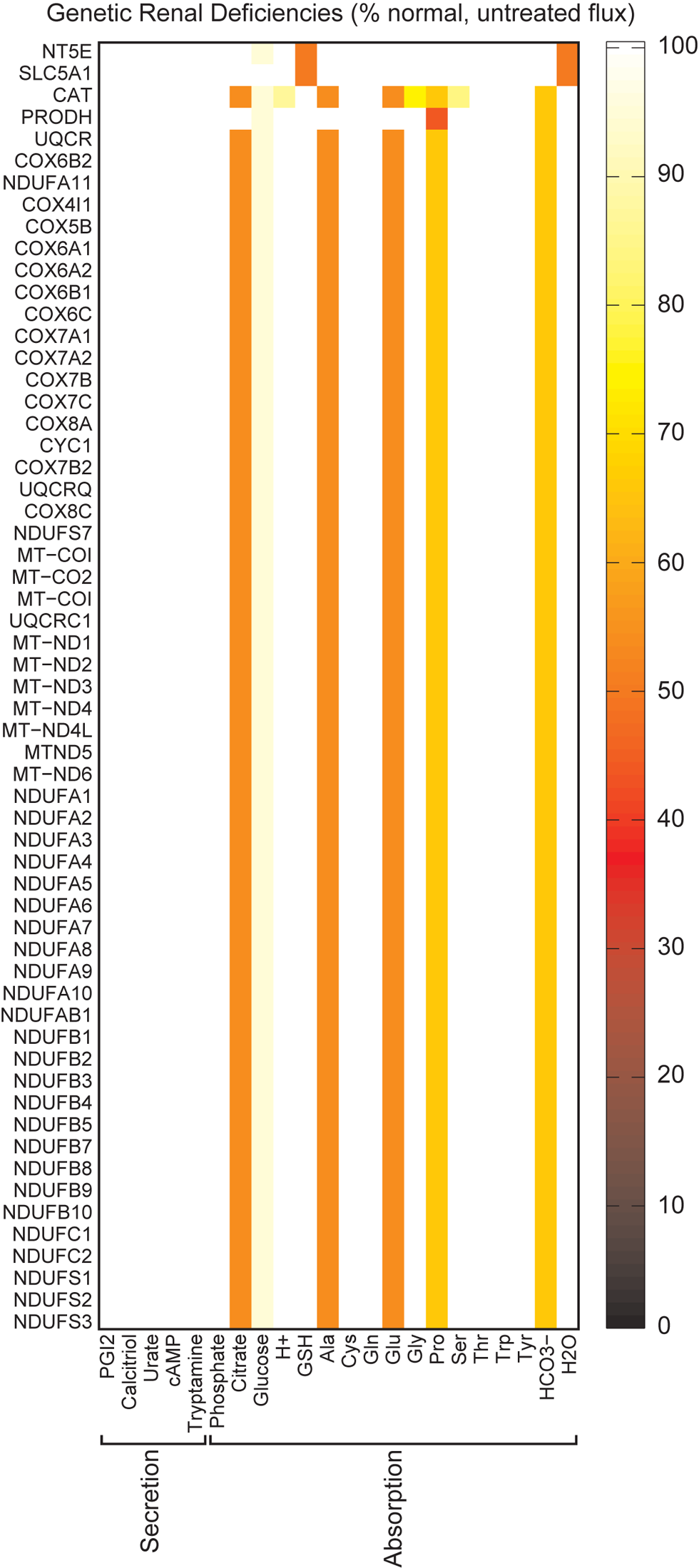

Supplement: Figure S1 — Genetic deficiencies causing renal metabolic disorders. Elements of the color matrix represent the percent of the maximum normal, untreated renal objective flux achievable by the drug-treated gene-deficient kidney model. The x-axis corresponds to individual renal objective functions, and the y-axis corresponds to the individual gene deficiencies represented by their official gene symbols. Metabolite abbreviations are defined in Table 1, and official gene symbols are defined in Table S4. Only the subset of renal objective functions for which a metabolic disorder was predicted is displayed. (0.53 MB TIF) [file pcbi.1000938.s002.tif]

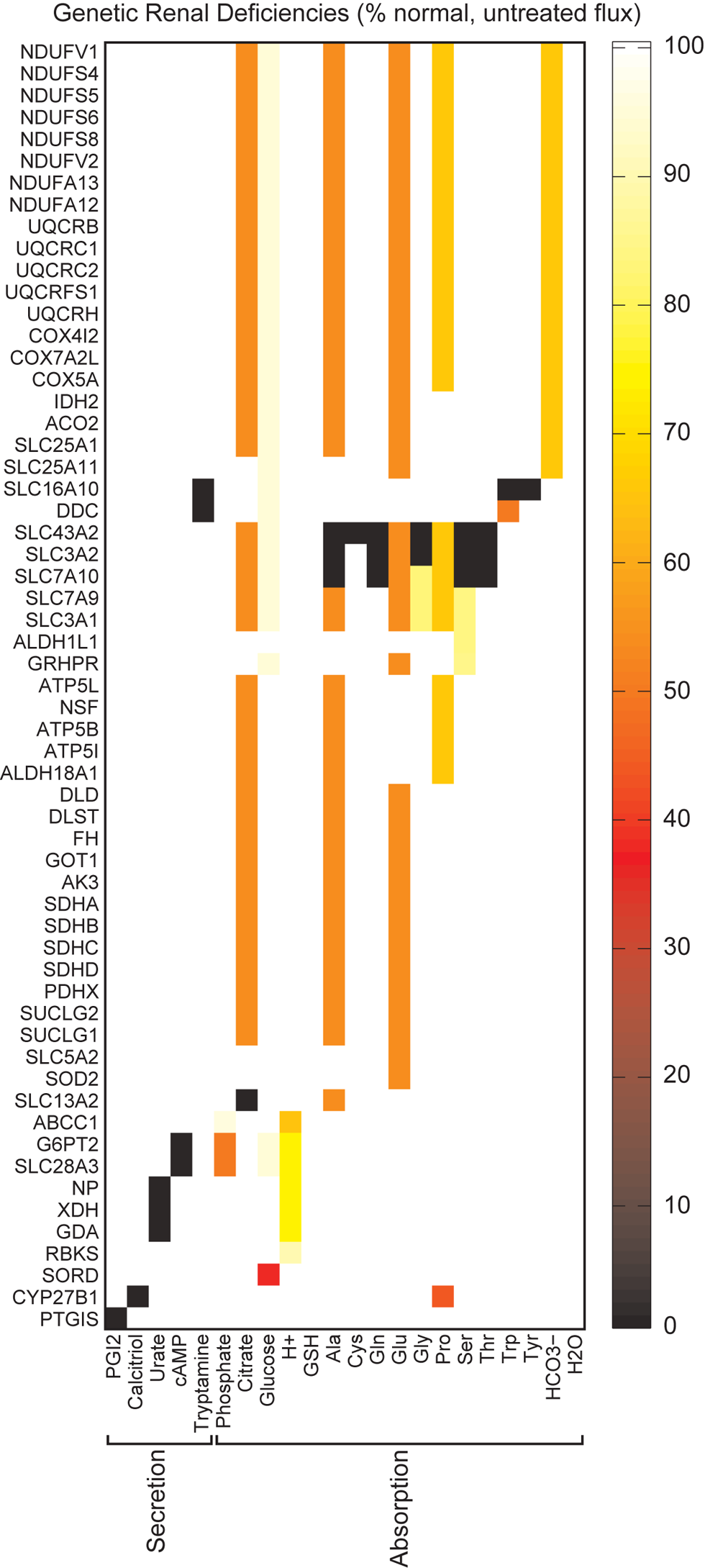

Supplement: Figure S2 — Genetic deficiencies causing renal metabolic disorders (continued). (0.48 MB TIF) [file pcbi.1000938.s003.tif]

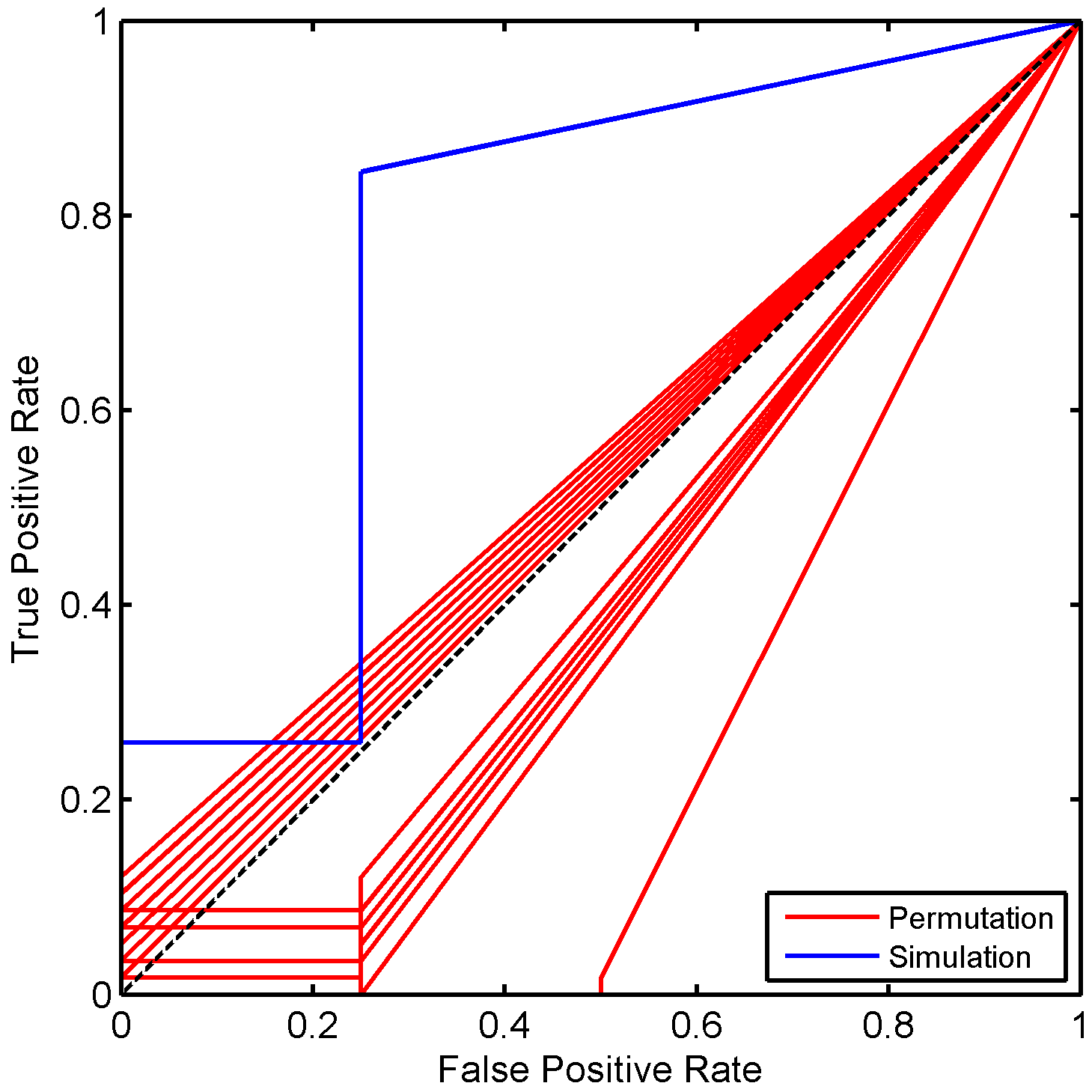

Supplement: Figure S3 — ROC curves for gene-deficient phenotype prediction. The blue line represents the analysis of the predictions of the model simulations presented in this study. The red lines represent the analysis of 100 different permutation trials. The dashed black line is the line y = x. (0.11 MB TIF) [file pcbi.1000938.s004.tif]

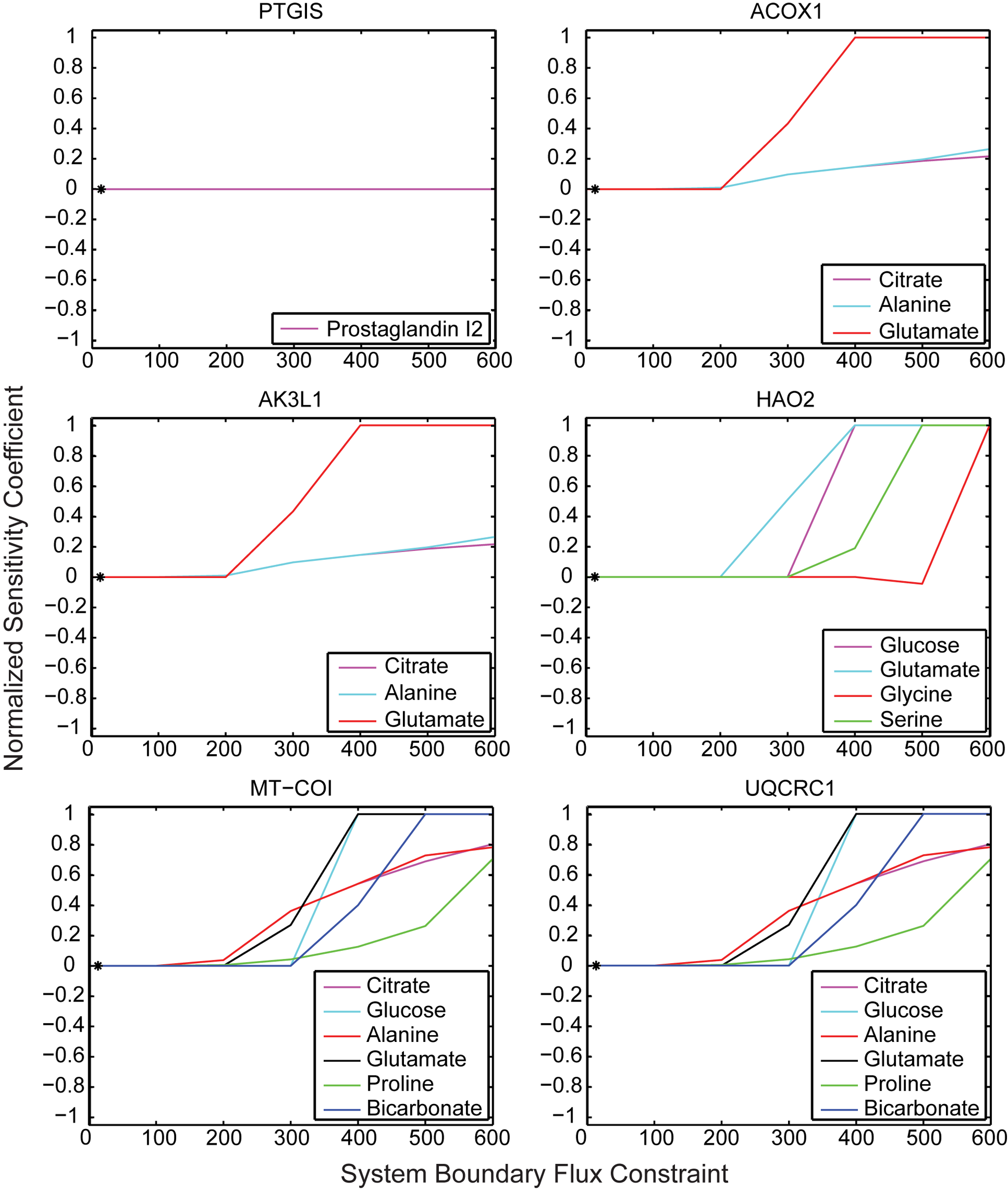

Supplement: Figure S4 — System boundary flux constraint sensitivity. Only those drug targets and renal functions are shown for which a deficient phenotype was predicted. The x-axis is in units of flux. The black star represents the base case which is presented as our primary result. (0.77 MB TIF) [file pcbi.1000938.s005.tif]

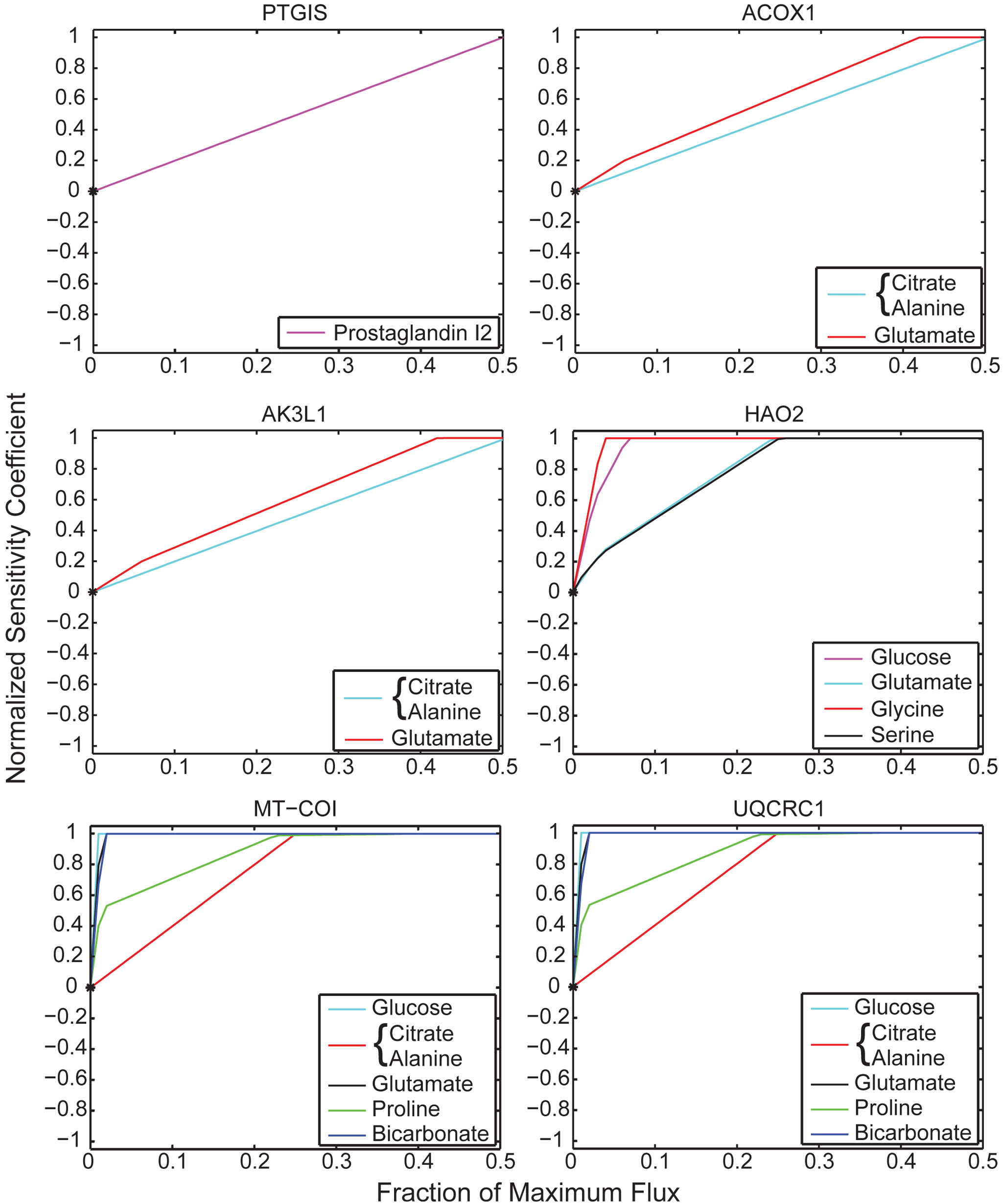

Supplement: Figure S5 — Degree of drug-induced inhibition sensitivity. Only those drug targets and renal functions are shown for which a deficient phenotype was predicted. The x-axis values correspond to the fraction of maximal enzymatic flux achievable in the untreated simulation, which represents the constraint placed on associated reactions for each simulation. The black star represents the base case which is presented as our primary result. (0.93 MB TIF) [file pcbi.1000938.s006.tif]
